# Depressive Symptomatology as a Predictor of Cognitive Impairment: Evidence from the Korean Longitudinal Study of Aging (KLOSA), 2006–2020

**DOI:** 10.3390/biomedicines11102713

**Published:** 2023-10-06

**Authors:** Seong-Uk Baek, Jin-Ha Yoon

**Affiliations:** 1 Department of Occupational and Environmental Medicine, Severance Hospital, Yonsei University College of Medicine, Seoul 03722, Republic of Korea; subaek@yuhs.ac; 2 The Institute for Occupational Health, Yonsei University College of Medicine, Seoul 03722, Republic of Korea; 3 Graduate School, Yonsei University College of Medicine, Seoul 03722, Republic of Korea; 4 Department of Preventive Medicine, Yonsei University College of Medicine, Seoul 03722, Republic of Korea

**Keywords:** neurodegenerative disease, cognitive health, dementia, epidemiology, depression, repeated measures analysis

## Abstract

Depressive symptoms are recognized as risk factors for cognitive impairment with intricate underlying biological mechanisms. We explored the link between depressive symptoms and cognitive impairment onset; we also assessed how this association is influenced by educational levels. This study included 5843 individuals aged ≥45 years, comprising 27,908 observations from 2006 to 2020. Based on repeated measurements of each participant, we estimated the association between depressive symptoms and cognitive impairment onset after a 2-year follow-up by using generalized estimating equations. The incidence rate was 9.4% among those individuals without depressive symptoms, which was in contrast with a rate of 21.0% among those individuals experiencing depressive symptoms. The odds ratio (OR) (95% confidence interval [CI]) for the association between depressive symptoms and cognitive impairment onset in the overall sample was 1.61 (1.47–1.76). This association was more pronounced among individuals with higher educational levels. Specifically, the OR (95% CI) of the association between depressive symptoms and cognitive impairment was highest among individuals with a college education (2.60 [1.78–3.81]), and the association was lowest among individuals with elementary or no education levels (1.45 [1.28–1.63]). Our findings highlight the idea that although individuals with higher educational backgrounds exhibit a diminished risk of cognitive impairment, the detrimental impacts of depressive symptoms on cognitive performance are particularly more pronounced within this group.

## 1. Introduction

Considering the anticipated trajectory of population aging, the burden of dementia is predicted to rapidly increase. A recent Global Burden of Disease study forecasted a tripling of dementia cases by 2050 compared with 2019 figures [1]. This impending rise is particularly striking in the Republic of Korea, which is a country grappling with one of the fastest aging population rates in the world, which has consequently led to a swift escalation in the economic burden associated with dementia and cognitive impairment [2]. Consequently, there is significant public health interest in exploring the risk factors for cognitive impairment and establishing preventative policies.

In the Republic of Korea, the prevalence of depressive symptoms has been consistently increasing, particularly among older adults [3,4]. Previous studies have indicated that depression or depressive pseudo-dementia serves as a risk factor for cognitive impairment [5,6,7,8], and this effect is potentially rooted in intricate biological mechanisms [9,10]. Previous studies have suggested that persistent depressive symptoms can decrease cerebral blood flow or lead to the secretion of excess glucocorticoids [9,10]. In conjunction with the cognitive reserve hypothesis [11], these multifaceted physiological shifts can erode cognitive reserves, thereby moderating the causal link between underlying neuropathology and its clinical manifestation as cognitive impairment [9]. The premise that depressive symptoms can heighten the susceptibility to cognitive impairment bears significance in the realm of dementia prevention, as these symptoms are often amenable to intervention.

Education is widely acknowledged as being one of the most influential factors in shaping cognitive impairment risk. A higher education level significantly reduces the risk of cognitive decline by increasing cognitive reserve [12,13]. For instance, a meta-analysis conducted by Xu et al. (2016) demonstrated an approximately 7% reduction in dementia risk for every additional year of education [14]. Several studies have focused on how educational level moderates the impact of cognitive impairment risk factors. For instance, previous studies showed that educational background can attenuate the detrimental effects of factors such as the *ApoE* e4 allele or cardiovascular risk on cognitive function [15,16]. Of particular significance, a study by Lee et al. (2018) demonstrated novel evidence suggesting that higher educational levels may mitigate the adverse influence of depression on cognitive impairment [17]. The authors contended that higher educational levels could mitigate the adverse effects of depressive symptoms on cognitive functioning by bolstering cognitive reserve. However, this study had several major limitations. First, their analysis was based on a cross-sectional design, thus neglecting the temporal sequence between depressive symptoms and cognitive impairment. Additionally, the study focused exclusively on a limited sample of female individuals, thereby constraining the generalizability of the findings.

Previous Korean studies have consistently indicated a connection between depressive symptoms and an increased risk of incident cognitive impairment [5,18]. However, these studies did not account for potential fluctuations in cognitive dysfunction and depression, as well as the lagged impact of depression [19]. Additionally, the moderating role of education in the relationship between depressive symptoms and cognitive impairment has not yet been investigated in a longitudinal framework. Therefore, by utilizing a 14-year cohort study with repeated measurements, we aimed to investigate the relationship between depressive symptoms and the risk of incident cognitive impairment among community-dwelling middle-aged or older adults in the Republic of Korea. Moreover, we endeavored to unveil how this relationship varies according to individual educational levels.

## 2. Materials and Methods

### 2.1. Study Sample

The study sample was derived from the first to eighth waves of the Korean Longitudinal Study of Ageing (KLoSA), which was conducted biannually by the Korea Employment Information Service (KEIS) between 2006 and 2020. The KLoSA aimed to include middle-aged and older adults aged ≥45 years who were residing in the Republic of Korea. In 2006, the KEIS employed systemic sampling, wherein enumeration districts were chosen as primary sampling units, and households within each district were selected as secondary sampling units. 

**Figure 1:**
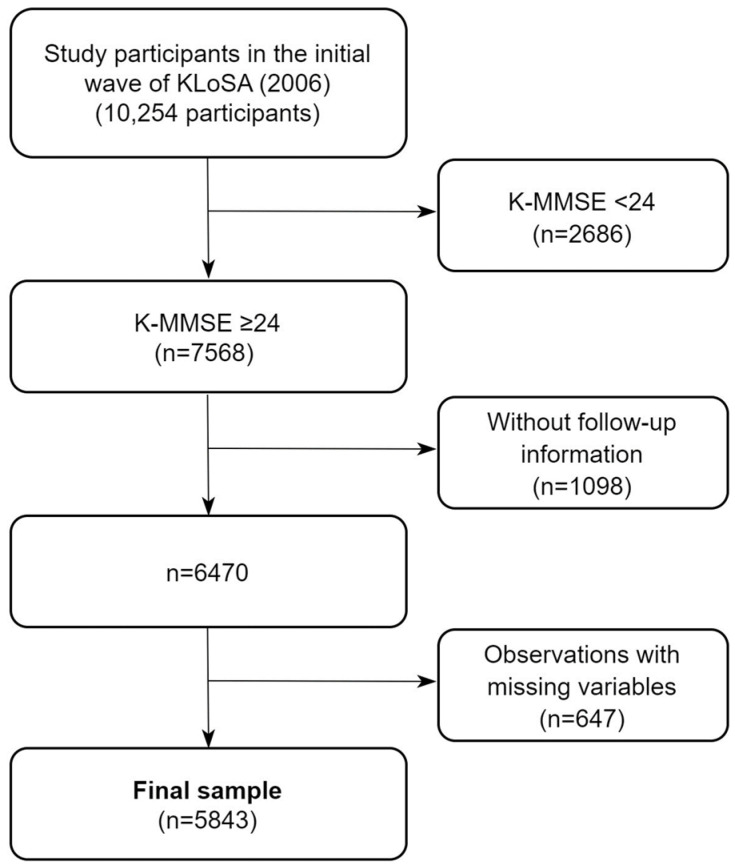
The flowchart of the selection of the study sample.

A flowchart of the study sample selection process is shown in Figure 1. A total of 10,254 middle-aged and older adults were included in the initial wave of the KLoSA (2006). Subsequently, we excluded individuals exhibiting cognitive impairment, which was defined as having a Korean-Mini Mental State Examination (K-MMSE) score of <24 in the initial wave, thus resulting in the exclusion of 2686 individuals. Further exclusions included 1098 individuals who did not participate in the second wave and subsequently lacked follow-up data. Next, observations with missing values were omitted, thus resulting in a final sample of 5843 individuals with 27,908 observations.

### 2.2. Data Availablity and Ethics Statement

The KLoSA data can be accessed at (https://survey.keis.or.kr/eng/index.jsp, accessed on 17 September 2023). The Institutional Review Board of Yonsei Health System has reviewed and approved this study (4-2022-1080).

### 2.3. Variables

#### 2.3.1. Exposure

Figure S1 shows the assumed causal relationship between depression and cognitive impairment onset, along with the confounding or moderating factors associated with this relationship. Depressive symptoms were assessed by using the Korean version of the Center of Epidemiologic Studies Depression Scale, 10-item version (CES-D-10). The CES-D-10 consists of 10 items that gauge the emotions and vitality of respondents over the preceding week. Each item is rated on a scale of 0–3. The total score ranges from 0–30, with higher scores indicating a greater presence of depressive symptoms. As suggested by a previous study, respondents with scores of 10 or above were classified as having depressive symptoms [20]. The reliability and validity of the CES-D-10 within a Korean context have been established through earlier investigations by Shin [21].

#### 2.3.2. Outcome

Cognitive function was assessed by using the K-MMSE. The K-MMSE consists of 11 items that measure cognitive performance, such as time and place orientations, attention, calculation, and recall. The total K-MMSE score ranges from 0–30, with higher scores indicating better cognitive performance. The validity of the K-MMSE for screening cognitive impairment was established in a previous study [22]. Following the cutoff scores that have been used in previous studies [18,23,24], K-MMSE scores <24 were considered to be indicative of cognitive impairment.

#### 2.3.3. Moderator

Educational attainment was categorized as “elementary school or below”, “middle school”, “high school”, and “college or above”. The categorization aligns with the structure of the Korean education system, which follows a unified 6–3–3–4 framework encompassing 6 years of elementary school, 3 years of middle school, 3 years of high school, and 4 years of college or university education [25].

#### 2.3.4. Confounders

We adjusted for several time-varying characteristics, and sex was adjusted accordingly. Age was treated as a continuous variable. Residential area was categorized as “metropolis”, “urban region”, and “rural region”. Marital status was categorized as “married”, “unmarried”, and “others (separated, divorced, or widowed)”. Income level was categorized based on the quintile values of total household income per survey year. Employment status was categorized as “employed” and “unemployed”. The adjusted comorbidities included hypertension, diabetes mellitus, cancer, pulmonary disease, liver disease, and cardiovascular disease, as they can influence both depression risk and cognitive function. The baseline K-MMSE score that was measured for each wave was adjusted in the regression models. All of the confounders (except for sex) were treated as time-varying covariates.

### 2.4. Statistical Analysis

**Figure 2:**
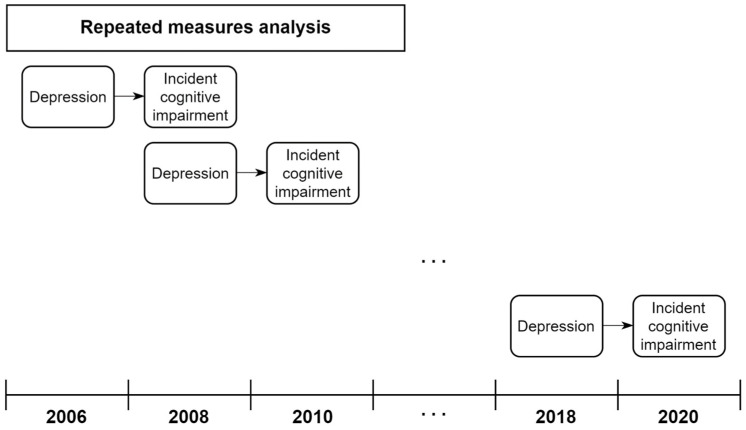
Study design of the repeated measures analysis.

For the descriptive analysis, we examined the characteristics of the study participants in the initial wave (2006) based on their depressive symptoms. The study design for the repeated-measures analysis is shown in Figure 2.

As depicted in the figure, in each year*_t_*, we explored how depressive symptoms in year*_t_* were associated with cognitive impairment onset in year*_t+2_* while also adjusting for the effect of time-varying confounders measured in each year*_t_*. Additionally, to clarify the temporal sequence, those with cognitive impairment (K-MMSE score < 24) in year*_t_* were excluded from the regression models. To conduct repeated measurements within each individual, a generalized estimating equation (GEE) approach was employed to estimate the population-averaged effect of depressive symptoms on subsequent cognitive impairment onset after a 2-year follow-up [26]. We first explored the association between depressive symptoms and cognitive impairment development in the overall sample in both the unadjusted (Model 1) and fully adjusted (Model 2) models. We then tested the moderating role of educational level on the association between depressive symptoms and cognitive impairment by introducing interaction terms (depressive symptoms × educational level) into Model 3. Upon confirming the moderating effect of education level, we performed a stratified analysis by educational level to explore how the association between depressive symptoms and cognitive impairment onset varies across educational subgroups. GEE models with a logit link function and an exchangeable working correlation matrix were employed to estimate odds ratios (ORs) and the corresponding 95% confidence intervals (CIs). All of the statistical analyses and visualizations were performed by using R software (version 4.2.3; R Foundation for Statistical Computing, Vienna, Austria), and GEE models were fitted by using the R function “geepack”.

### 2.5. Sensitivity Analysis

Missing values were addressed by using multiple imputations with a chained equation (MICE). Twenty datasets without missing values were generated, and the estimates were combined by utilizing Rubin’s rule. MICE was conducted by using the R package “mice”. Sensitivity analysis results are presented in the Supplementary Materials.

## 3. Results

Table 1 presents the characteristics of the study sample during the initial wave. The sample consists of 2866 (49.1%) male and 2977 (50.9%) female participants. A total of 857 (14.7%) patients experienced depressive symptoms. The group exhibiting depressive symptoms had a higher proportion of females, older individuals, those with lower educational and income levels, those who were unmarried, those who were not engaged in economic activities, those with comorbidities, and those with lower initial K-MMSE scores.

Table 2 presents the incidence rate of cognitive impairment onset according to the study characteristics. The incidence rate of cognitive impairment onset was 9.4% for those without depressive symptoms and 21.0% for those with depressive symptoms. Additionally, women, older individuals, those with lower educational attainment, and individuals with lower income levels were more likely to develop cognitive impairment.

Table 3 shows the factors associated with the development of cognitive impairment after a 2-year follow-up. In the fully adjusted model (Model 2), the OR (95% CI) for the association between depressive symptoms and incident cognitive impairment was 1.61 (1.47–1.76). In Model 3, there were positive interactions between depressive symptoms and high school education (OR [95% CI]: 1.31 [1.05–1.64]; *p* = 0.018) and college education (OR [95% CI]: 1.73 [1.19–2.52]; *p* = 0.004) on incident cognitive impairment risk. 

**Figure 3:**
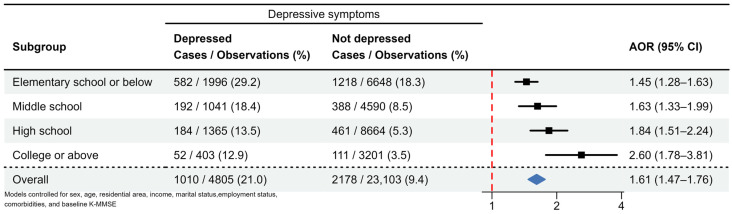
Stratified analyses of the impact of depressive symptoms on the risk of cognitive impairment by educational level. (AOR: Adjusted odds ratio; CI: confidence intervals).

In the stratified analysis (Figure 3), the adjusted OR (95% CI) of the relationship between depressive symptoms and incident cognitive impairment was 1.45 (1.28–1.63) among individuals with elementary school education, 1.63 (1.33–1.99) among those with middle school education, 1.84 (1.51–2.24) among those with high school education, and 2.60 (1.78–3.81) among those with college education.

In the sensitivity analysis, we also confirmed positive interactions between depressive symptoms and high school or college education on the risk of cognitive impairment onset in the overall sample (results not shown). Table S1 shows that the association between depressive symptoms and cognitive impairment onset was greater among the subgroups with higher educational levels, thus supporting the findings of the main analyses.

## 4. Discussion

Our study revealed that individuals experiencing depressive symptoms had a higher likelihood of cognitive impairment development than those without such symptoms. Moreover, this relationship displayed variability in accordance with the educational attainment of the individual. Notably, those with higher educational levels generally exhibited a reduced risk of incident cognitive impairment over a 2-year follow-up period. However, the amplification in the odds of cognitive impairment onset linked with depressive symptoms was particularly pronounced in individuals with higher educational achievements. These findings remained robust even after adjusting for demographic and socioeconomic characteristics, as well as baseline cognitive function. Consequently, our findings highlight the necessity of monitoring depressive symptoms as a means to safeguard the cognitive function of individuals with advanced educational backgrounds.

Our findings reconfirmed several well-established risk factors for cognitive impairment that have been consistently observed in the previous literature, including advanced age, low educational level, low income level, and low baseline cognitive function. Notably, although the overall incidence rate among all of the observations was 11.4%, this rate significantly increased within the older population and among groups with lower baseline MMSE scores. Considering that these high-risk groups constituted the majority of cases, it is imperative to closely monitor the cognitive function of these vulnerable individuals.

Our findings align with the results of previous studies and restate the positive correlation that has been documented between depressive symptoms and the likelihood of developing incident cognitive impairment [7,8,27,28]. Although depressive symptoms are frequently regarded as being prodromal indicators of clinical dementia, mounting evidence suggests that depressive symptoms in later stages of life may act as an independent risk factor for cognitive impairment [29]. Previous Korean studies have consistently demonstrated that depressive symptoms are associated with the development of cognitive impairment during advanced age [5,30,31]. Vascular diseases and elevated glucocorticoid levels have been postulated as representing a causal link between depression and cognitive impairment [10]. These alterations may contribute to an increased cognitive impairment risk by inducing hippocampal atrophy, inflammatory changes, or elevated amyloid plaques [8].

The primary finding of our study demonstrates that the impact of depressive symptoms on cognitive impairment onset may be more pronounced among individuals with higher educational levels. This contradicts the conclusions drawn by Lee et al., who proposed a potential protective role of a strong educational background against the detrimental effect of depressive symptoms on memory performance [17]. Our study possesses the advantage of incorporating a longitudinal design, thus enhancing its generalizability across diverse populations. Additionally, in contrast to the educational level categorization that was utilized in the previous study as “uneducated” versus “elementary school or higher education” [17] (which limited the assessment of the influence of higher education, including high school or college/university education, on the effect of depressive symptoms), our research delved into the nuanced effects of education within more sophisticated strata. This approach produces practical benefits in terms of implications, as it accounts for the overall evolution of educational attainment in the Republic of Korea over the recent decades [32].

Multiple explanations can be postulated to elucidate why the impact of depression on cognitive performance holds particular significance for individuals with advanced education. According to the cognitive reserve theory, individuals with higher educational levels experience benefits from an augmented cognitive reserve cultivated through engagement in intellectually stimulating activities and tasks throughout their lives [33,34,35]. Depressive symptoms tend to impede the cognitive attributes of swift cognitive processing and sustained concentration, which are attributes particularly relevant to individuals who have honed such skills over extended periods of education [36]. Moreover, depressive symptoms often impede active social or leisure engagement, consequently contributing to cognitive impairment [37]. Considering that the cognitive reserve of individuals with higher education often reaps advantages from rich activity engagement [38], the adverse effects of depressive symptoms on cognitive function may be particularly pronounced among this subgroup. Indeed, a prior study conducted by Potvin et al. showed that elevated cortisol levels, indicative of heightened stress, were associated with an increased incident cognitive impairment risk, primarily among individuals with high educational levels [39]. A more comprehensive exploration employing hormonal or neural activity measurements is required to elucidate the underlying biological mechanisms that underscore the heightened susceptibility of highly educated individuals to the impacts of depressive symptoms on cognitive function. 

Our study exhibited the following clinical implications. First, our findings suggest that the promotion of the mental health of middle-aged or older adults is crucial for maintaining their cognitive well-being. Therefore, policy efforts are needed to prevent depressive symptoms in the older population. Second, our study suggests that older adults with depression should be closely monitored for both their cognitive function and depressive symptoms. Finally, our results highlight the idea that depressive symptoms are an important risk factor for cognitive impairment, even in highly educated groups with a rich baseline cognitive reserve. Therefore, programs and services aimed at promoting mental health and managing cognitive function should be considered to benefit individuals of all educational backgrounds.

This study was subject to several limitations. First, due to the observational nature of the study design, we cannot definitively establish a causal relationship between depressive symptoms, educational level, and cognitive impairment. For instance, the absence of data on potential confounders, such as the *ApoE* e4 allele and family history, introduced the possibility of unmeasured confounding factors. Second, although the K-MMSE and CES-D-10 are widely used and validated measurements of cognitive impairment and depression, respectively, they were originally developed for screening purposes. Therefore, it is imperative that future research explores the relationship between clinically diagnosed depression and dementia to validate our findings. Third, during the process of sample selection, a notable number of missing values and non-participating participants in the follow-up survey were identified. Although the sensitivity analysis using multiple imputations demonstrated the robustness of our findings, it is important to note that multiple imputations rely on the assumption of missing data being random. If this assumption is violated, there remains a potential for biased estimations [40].

## 5. Conclusions

In our investigation, we observed a significant association between depressive symptoms and a heightened vulnerability to cognitive impairment onset, a link that exhibited noteworthy prominence among individuals with higher educational achievements. Although individuals with higher educational attainments demonstrated a reduction in the risk of developing cognitive impairment, the detrimental influence of depressive symptoms on cognitive health was more prominently manifested within this specific demographic. Our findings highlight the importance of addressing depressive symptoms in older adults with high educational attainment as a preventative measure against the onset of cognitive impairment.

## Figures and Tables

**Table 1:** Characteristics of the study participants in the initial wave of KLoSA, 2006 (K-MMSE: Korean Mini-Mental State Exam).

	Overall	Depressive Symptoms		*p* Value *
		Yes	No	
	n = 5843	n = 857	n = 4986	
**Sex**				<0.001
Male	2866 (49.1%)	343 (40.0%)	2523 (50.6%)	
Female	2977 (50.9%)	514 (60.0%)	2463 (49.4%)	
**Age**				<0.001
Mean (standard deviation)	58.4 (9.4)	61.1 (9.9)	58.0 (9.2)	
**Educational level**				<0.001
Elementary school or below	2059 (35.2%)	478 (55.8%)	1581 (31.7%)	
Middle school	1122 (19.2%)	152 (17.7%)	970 (19.5%)	
High school	1957 (33.5%)	185 (21.6%)	1772 (35.5%)	
College or above	705 (12.1%)	42 (4.9%)	663 (13.3%)	
**Residential area**				0.036
Metropolis	2625 (44.9%)	371 (43.3%)	2254 (45.2%)	
Urban region	1889 (32.3%)	262 (30.6%)	1627 (32.6%)	
Rural region	1329 (22.7%)	224 (26.1%)	1105 (22.2%)	
**Income level**				<0.001
Q1	886 (15.2%)	188 (21.9%)	698 (14.0%)	
Q2	1045 (17.9%)	232 (27.1%)	813 (16.3%)	
Q3	1617 (27.7%)	239 (27.9%)	1378 (27.6%)	
Q4	1029 (17.6%)	107 (12.5%)	922 (18.5%)	
Q5	1266 (21.7%)	91 (10.6%)	1175 (23.6%)	
**Marital status**				<0.001
Married	5024 (86.0%)	597 (69.7%)	4427 (88.8%)	
Unmarried	769 (13.2%)	247 (28.8%)	522 (10.5%)	
Others	50 (0.9%)	13 (1.5%)	37 (0.7%)	
**Employment status**				<0.001
Employed	2845 (48.7%)	294 (34.3%)	2551 (51.2%)	
Unemployed	2998 (51.3%)	563 (65.7%)	2435 (48.8%)	
**Hypertension**				<0.001
Yes	1407 (24.1%)	266 (31.0%)	1141 (22.9%)	
No	4436 (75.9%)	591 (69.0%)	3845 (77.1%)	
**Diabetes mellitus**				<0.001
Yes	609 (10.4%)	135 (15.8%)	474 (9.5%)	
No	5234 (89.6%)	722 (84.2%)	4512 (90.5%)	
**Cancer**				<0.001
Yes	130 (2.2%)	42 (4.9%)	88 (1.8%)	
No	5713 (97.8%)	815 (95.1%)	4898 (98.2%)	
**Pulmonary disease**				<0.001
Yes	95 (1.6%)	28 (3.3%)	67 (1.3%)	
No	5748 (98.4%)	829 (96.7%)	4919 (98.7%)	
**Liver disease**				
Yes	95 (1.6%)	20 (2.3%)	75 (1.5%)	0.135
No	5748 (98.4%)	837 (97.7%)	4911 (98.5%)	
**Cardiovascular disease**				<0.001
Yes	233 (4.0%)	62 (7.2%)	171 (3.4%)	
No	5610 (96.0%)	795 (92.8%)	4815 (96.6%)	
**Baseline K-MMSE**				<0.001
Mean (standard deviation)	27.9 (1.9)	27.2 (2.0)	28.0 (1.8)	

* Chi-square test or *t*-test.

**Table 2:** Incidence rate of cognitive impairment according to study characteristics throughout the observational period in the KLoSA, 2006–2020.

	Cognitive Impairment Onset		*p* Value *
	Yes	No	
	n = 3188	n = 24,720	
**Depressive symptoms**			<0.001
Not depressed	2,178 (9.4%)	20,925 (90.6%)	
Depressed	1010 (21.0%)	3795 (79.0%)	
**Sex**			<0.001
Male	1336 (9.8%)	12,338 (90.2%)	
Female	1852 (13.0%)	12,382 (87.0%)	
**Age**			<0.001
45–54	272 (4.4%)	5888 (95.6%)	
55–64	816 (7.4%)	10,152 (92.6%)	
65–74	1313 (16.7%)	6546 (83.3%)	
≥75	787 (26.9%)	2134 (73.1%)	
**Educational level**			<0.001
Elementary school or below	1800 (20.8%)	6844 (79.2%)	
Middle school	580 (10.3%)	5051 (89.7%)	
High school	645 (6.4%)	9384 (93.6%)	
College or above	163 (4.5%)	3441 (95.5%)	
**Residential area**			<0.001
Metropolis	1229 (9.9%)	11,157 (90.1%)	
Urban region	986 (10.8%)	8146 (89.2%)	
Rural region	973 (15.2%)	5417 (84.8%)	
**Income level**			<0.001
Q1	847 (23.0%)	2834 (77.0%)	
Q2	824 (15.3%)	4568 (84.7%)	
Q3	675 (9.9%)	6115 (90.1%)	
Q4	498 (7.9%)	5802 (92.1%)	
Q5	344 (6.0%)	5401 (94.0%)	
**Marital status**			<0.001
Married	2442 (10.2%)	21,522 (89.8%)	
Unmarried	714 (19.1%)	3026 (80.9%)	
Others	32 (15.7%)	172 (84.3%)	
**Employment status**			<0.001
Employed	1084 (7.8%)	12,891 (92.2%)	
Unemployed	2104 (15.1%)	11,829 (84.9%)	
**Hypertension**			<0.001
Yes	1377 (15.3%)	7601 (84.7%)	
No	1811 (9.6%)	17,119 (90.4%)	
**Diabetes mellitus**			<0.001
Yes	599 (15.6%)	3240 (84.4%)	
No	2589 (10.8%)	21,480 (89.2%)	
**Cancer**			<0.001
Yes	175 (14.7%)	1014 (85.3%)	
No	3013 (11.3%)	23,706 (88.7%)	
**Pulmonary disease**			<0.001
Yes	110 (19.9%)	443 (80.1%)	
No	3078 (11.3%)	24,277 (88.7%)	
**Liver disease**			<0.001
Yes	85 (12.8%)	581 (87.2%)	
No	3103 (11.4%)	24,139 (88.6%)	
**Cardiovascular disease**			<0.001
Yes	292 (17.1%)	1420 (82.9%)	
No	2896 (11.1%)	23,300 (88.9%)	
**Baseline K-MMSE**			<0.001
24–26	1614 (25.1%)	4820 (74.9%)	
27–28	845 (10.8%)	6964 (89.2%)	
29–30	729 (5.3%)	12,936 (94.7%)	

* Chi-square test or *t*-test.

**Table 3:** Association between depressive symptoms and cognitive impairment onset after a 2-year follow-up (OR: odds ratio; CI; confidence Interval).

	Model 1		Model 2		Model 3	
	OR (95% CI)	*p* Value	OR (95% CI)	*p* Value	OR (95% CI)	*p* Value
**Depressive symptoms**						
Not depressed	Reference		Reference		Reference	
Depressed	2.17 (2.00–2.36)	<0.001	1.61 (1.47–1.76)	<0.001	1.43 (1.27–1.62)	<0.001
**Sex**						
Male			Reference		Reference	
Female			1.22 (1.10–1.34)	<0.001	1.21 (1.10–1.34)	<0.001
**Age**						
Continuous variable			1.06 (1.05–1.06)	<0.001	1.05 (1.05–1.06)	<0.001
**Educational level**						
Elementary school or below			Reference		Reference	
Middle school			0.73 (0.64–0.82)	<0.001	0.69 (0.60–0.79)	<0.001
High school			0.56 (0.50–0.63)	<0.001	0.52 (0.45–0.59)	<0.001
College or above			0.40 (0.33–0.48)	<0.001	0.34 (0.27–0.43)	<0.001
**Residential area**						
Metropolis			Reference		Reference	
Urban region			1.14 (1.03–1.27)	0.012	1.13 (1.02–1.26)	0.017
Rural region			1.28 (1.14–1.42)	<0.001	1.27 (1.14–1.42)	<0.001
**Income level**						
Q1			Reference		Reference	
Q2			0.87 (0.77–0.97)	0.015	0.87 (0.77–0.97)	0.014
Q3			0.78 (0.68–0.88)	<0.001	0.77 (0.68–0.88)	<0.001
Q4			0.82 (0.71–0.94)	0.006	0.82 (0.71–0.94)	0.004
Q5			0.86 (0.73–1.01)	0.067	0.86 (0.73–1.01)	0.071
**Marital status**						
Married			Reference		Reference	
Unmarried			1.04 (0.92–1.17)	0.552	1.04 (0.92–1.17)	0.529
Others			1.86 (1.14–3.03)	0.013	1.83 (1.13–2.94)	0.013
**Employment status**						
Employed			Reference		Reference	
Unemployed			1.20 (1.09–1.32)	<0.001	1.20 (1.09–1.32)	<0.001
**Hypertension**						
Yes			1.06 (0.97–1.16)	0.222	1.06 (0.96–1.16)	0.244
No			Reference		Reference	
**Diabetes mellitus**						
Yes			1.09 (0.97–1.23)	0.156	1.09 (0.97–1.23)	0.150
No			Reference		Reference	
**Cancer**						
Yes			1.08 (0.90–1.31)	0.406	1.08 (0.89–1.30)	0.445
No			Reference		Reference	
**Pulmonary disease**						
Yes			1.32 (1.05–1.66)	0.017	1.32 (1.05–1.66)	0.017
No			Reference		Reference	
**Liver disease**						
Yes			1.01 (0.78–1.30)	0.962	1.01 (0.78–1.30)	0.929
No			Reference		Reference	
**Cardiovascular disease**						
Yes			1.07 (0.92–1.26)	0.381	1.08 (0.92–1.26)	0.367
No			Reference		Reference	
**Baseline K-MMSE**						
Continuous variable			0.76 (0.75–0.78)	<0.001	0.76 (0.75–0.78)	<0.001
**Depressive symptoms ×** **Educational levels**						
Depressed × Middle school					1.18 (0.94–1.48)	0.160
Depressed × High school					1.31 (1.05–1.64)	0.018
Depressed × College or above					1.73 (1.19–2.52)	0.004

## Data Availability

The KLoSA data are available at (https://survey.keis.or.kr/eng/index.jsp, accessed on 17 September 2023).

## Supplementary Materials

The following supporting information can be downloaded at https://www.mdpi.com/article/10.3390/biomedicines11102713/s1. Figure S1: Assumed causal relationship between depression and cognitive impairment. Table S1: Results of the sensitivity analyses based on the imputed datasets (AOR: Adjusted odds ratio; CI: confidence intervals).
