# Supplementary material for: Depressive Symptomatology as a Predictor of Cognitive Impairment: Evidence from the Korean Longitudinal Study of Aging (KLOSA), 2006–2020
Source: Biomedicines. 2023 Oct 6;11(10):2713. doi: 10.3390/biomedicines11102713 (PMC10604701; doi:10.3390/biomedicines11102713)
Supplement: Supplementary file 1 [file biomedicines-11-02713-s001.zip › biomedicines-2637579-supplementary.pdf]

Depressive Symptomatology as a Predictor of Cognitive Impairment: Evidence from the Korean Longitudinal Study of Aging (KLOSA), 2006–2020

Supplementary Materials

Seong-Uk Baek [1,2,3] and Jin-Ha Yoon [2,4,*]

[1] Department of Occupational and Environmental Medicine, Severance Hospital, Yonsei University College of Medicine, Seoul 03722, Republic of Korea

[2] The Institute for Occupational Health, Yonsei University College of Medicine, Seoul 03722, Republic of Korea

[3] Graduate School, Yonsei University College of Medicine, Seoul 03722, Republic of Korea

[4] Department of Preventive Medicine, Yonsei University College of Medicine, Seoul 03722, Republic of Korea

* Correspondence: flyinyou@yuhs.ac

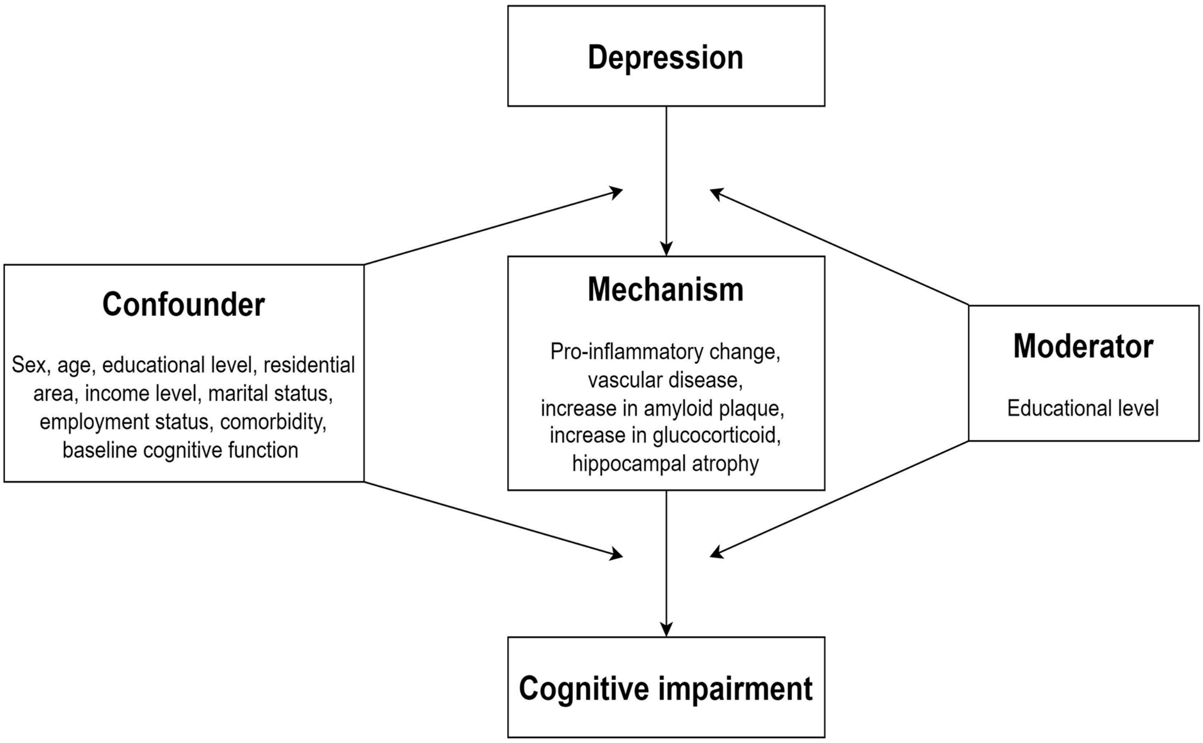

Figure S1. Assumed causal relationship between depression and cognitive impairment.

Table S1. Sensitivity analysis results based on the imputed datasets (AOR: Adjusted Odds Ratio; CI: Confidence Intervals).

	AOR (95% CI)	*p* Value
**Overall**	1.62 (1.49–1.76)	<0.001
**Subgroup**		
Elementary school or below	1.45 (1.29–1.62)	<0.001
Middle school	1.68 (1.39–2.03)	<0.001
High school	1.83 (1.52–2.22)	<0.001
College or above	2.39 (1.67–3.41)	<0.001
